# The association between resting‐state functional magnetic resonance imaging and aortic pulse‐wave velocity in healthy adults

**DOI:** 10.1002/hbm.24934

**Published:** 2020-02-07

**Authors:** Ahmad Hussein, Jacob L. Matthews, Catriona Syme, Christopher Macgowan, Bradley J. MacIntosh, Zahra Shirzadi, Zdenka Pausova, Tomáš Paus, J. Jean Chen

**Affiliations:** ^1^ Rotman Research Institute, Baycrest Health Sciences Toronto Canada; ^2^ SickKids Hospital Toronto Canada; ^3^ Department of Physiology University of Toronto Toronto Canada; ^4^ Department of Medical Biophysics University of Toronto Toronto Canada; ^5^ Physical Sciences Platform Sunnybrook Research Institute Toronto Canada; ^6^ Bloorview Research Institute Holland Bloorview Kids Rehabilitation Hospital Toronto Canada

**Keywords:** aging, aortic stiffness, arterial stiffening, blood pressure, blood‐oxygenation level dependent signal (BOLD), BOLD signal amplitude, functional connectivity, functional MRI (fMRI), phase‐contrast imaging, pulse wave velocity, resting‐state fMRI

## Abstract

Resting‐state functional magnetic resonance imaging (rs‐fMRI) is frequently used to study brain function; but, it is unclear whether BOLD‐signal fluctuation amplitude and functional connectivity are associated with vascular factors, and how vascular‐health factors are reflected in rs‐fMRI metrics in the healthy population. As arterial stiffening is a known age‐related cardiovascular risk factor, we investigated the associations between aortic stiffening (as measured using pulse‐wave velocity [PWV]) and rs‐fMRI metrics. We used cardiac MRI to measure aortic PWV (an established indicator of whole‐body vascular stiffness), as well as dual‐echo pseudo‐continuous arterial‐spin labeling to measure BOLD and CBF dynamics simultaneously in a group of generally healthy adults. We found that: (1) higher aortic PWV is associated with lower variance in the resting‐state BOLD signal; (2) higher PWV is also associated with lower BOLD‐based resting‐state functional connectivity; (3) regions showing lower connectivity do not fully overlap with those showing lower BOLD variance with higher PWV; (4) CBF signal variance is a significant mediator of the above findings, only when averaged across regions‐of‐interest. Furthermore, we found no significant association between BOLD signal variance and systolic blood pressure, which is also a known predictor of vascular stiffness. Age‐related vascular stiffness, as measured by PWV, provides a unique scenario to demonstrate the extent of vascular bias in rs‐fMRI signal fluctuations and functional connectivity. These findings suggest that a substantial portion of age‐related rs‐fMRI differences may be driven by vascular effects rather than directly by brain function.

## 1 INTRODUCTION

Arterial stiffening is associated with higher risk for stroke and cardiovascular disease (Gavish & Izzo, 2016). A healthy aorta (and carotid artery) acts as an elastic buffer and converts the systolic inflow into a smoother, continuous outflow to the brain (and other organs). This so‐called “Windkessel effect” is impared in stiff arteries (Belz, 1995), leading to increased pulsatile flow at the brain, which is a potential risk factor of cerebral small‐vessel damage. (van Elderen et al., 2010). The speed of the pressure wave is known as pulse wave velocity (PWV) and is the gold standard marker of arterial stiffness (Mattace‐Raso et al., 2007; Mitchell et al., 2011). In essence, PWV depends on arterial pressure and the elastic properties of arterial walls. As the pulsatility of the heart, it is absorbed in part through intracranial compliance and further enabled by the existence of the cerebrospinal fluid (CSF) in the subarachnoid space and the ventricular system (Wagshul, Eide, & Madsen, 2011). However, with arterial stiffening, the pulse waves may not be adequately absorbed, resulting in potential damage to brain tissue and vessels (Mitchell et al., 2011). Specifically, higher PWV has been associated with an increased incidence of cerebral small‐vessel disease (van Elderen et al., 2010), as well as reduced perfusion of white matter (WM) (Tarumi, Shah, Tanaka, & Haley, 2011).

Steward et al. showed that PWV is negatively correlated with the amplitude of the blood‐oxygenation level dependent (BOLD) functional magnetic resonance imaging (fMRI) response measured in a working‐memory task in healthy middle‐aged individuals (Steward et al., 2014). This provided the first evidence of the impact of arterial stiffening on the BOLD task response. However, resting‐state fMRI (rs‐fMRI) has been more widely used in studying brain function, particularly dominating the study of cognitive aging. The rs‐fMRI signal fluctuation amplitude and corresponding functional connectivity are both driven by vascular factors including cerebrovascular reactivity (CVR) and cerebral blood flow (CBF) (Chu, Golestani, Kwinta, Khatamian, & Chen, 2018; Golestani, Kwinta, Strother, Khatamian, & Chen, 2016). Moreover, it is well documented that both the amplitude of the BOLD response (Ances et al., 2009) and the variance of resting‐state BOLD signal decrease with increasing age (Grady & Garrett, 2014). The same is true for functional connectivity measured in resting‐state data (Brier et al., 2013). Increased carotid PWV in aging has been associated with reduced blood flow in aging (Fisher et al., 2013; Jefferson et al., 2018; Kroner et al., 2013) as well as higher blood pressure (Sawabe et al., 2005). Arterial stiffening has also been associated with reduced CVR (Flück et al., 2014). Therefore, arterial stiffening can embody a number of vascular changes in aging; but, it is still unclear how PWV is associated with different rs‐fMRI measures, which are based mainly on changes in blood oxygenation. To date, the only link between functional connectivity and PWV can be gleaned from a study using a mouse model of arterial stiffening; Guevara et al. showed an inverse relationship between PWV and functional connectivity as measured using functional near‐infrared spectroscopy (Guevara, Sadekova, Girouard, & Lesage, 2013). Thus, it is still unclear if and how elevated PWV contributes to inter‐individual variations in rs‐fMRI metrics in healthy aging humans.

In this study, we investigate the associations between aortic PWV (measured between aortic arch and abdominal aorta) and rs‐fMRI metrics, including signal‐fluctuation amplitude and functional connectivity, in healthy adults. As systemic (whole‐body) arterial stiffness is known to be reflected in the flow pattern of major cerebral arteries (Xu et al., 2012), we used cardiac MRI to measure aortic PWV, which is a good approximation of carotid stiffness (Kröner et al., 2014; Nagai et al., 1999). Based on the literature outlined earlier (Chu et al., 2018; Golestani et al., 2016; Guevara et al., 2013; Steward et al., 2014), we hypothesize that (1) as arterial stiffening leads to CVR reduction, the amplitude of the BOLD signal fluctuations is inversely associated with PWV; (2) as arterial stiffening is a hallmark of aging, these fMRI‐related associations are similar to the effect of age; (3) as arterial stiffening is associated with CBF and CVR reduction, BOLD‐based functional connectivity is inversely associated with PWV. Furthermore, we investigate the roles of (systemic) blood pressure and contribution of local cerebral blood flow in these relationships.

## 2 MATERIALS AND METHODS

### 2.1 Participants and anthropometric assessments

The sample consisted of parents (ages 43–67) and their adult offspring (ages 19–39) for a total of 59 participants (25 parents ages 44–67 [9 males/16 females], 24 offspring ages 18–39 [7 males/17 females]) (Table 1). Participants were recruited through the Baycrest Participants Database, consisting of individuals from the Baycrest and local communities. The study was approved by the Research Ethics Board of Baycrest, and the experiments were performed with the understanding and written consent of each participant. As this was a pilot study for a larger, population‐based multi‐generational study with the intention of representing the general population. The exclusion criteria are: (1) English‐language deficiency; (2) medical disorders, including diabetes (Type I), systemic rheumatologic illnesses, systemic malignancy, congenital heart defect, aneurysm, and arthritis; (3) brain disorders, including seizures, meningitis, brain tumors, untreated psychosis, muscular dystrophy, myotonic dystrophy, and a history of closed head injury with loss of consciousness >30 min; (4) developmental disorders, including autism, phenylketonuria, Asperger syndrome, hearing impairment, and visual impairment; (5) MRI contraindications, including metal implants/fragments, pacemakers, and claustrophobia.

**Table 1:** Demographic information for the participants. The data indicates that both groups are generally healthy

Parameter	Parent group	Offspring group
Age (yr)	55.0 ± 6.5	25.4 ± 5.8
Female/male ratio	0.64	0.70
Height (cm)	169.7 ± 8.2	171.3 ± 10.9
Mean BP (mmHg)	84.8 ± 8.5	82.7 ± 8.2
Weight (kg)	77.1 ± 15.7	69.8 ± 14.3
Body‐mass index	27.0 ± 5.8	24.0 ± 3.8
Mean systolic BP (mmHg)	122.5 ± 13.2	119.8 ± 14.8
Mean diastolic BP (mmHg)	67.5 ± 8.3	65.8 ± 5.8
Pulse pressure (mmHg)	55.0 ± 11.7	53.2 ± 10.8
Heart rate (beats/min)	69.0 ± 11.3	69.3 ± 8.5

Participants underwent a full cardiovascular and cognitive assessment to ensure healthy brain function. The cardiovascular assessment provided the systolic blood pressure (SBP) and diastolic blood pressure (DBP), measured by Finometer (FMS Finapres, Amsterdam, The Netherlands) before the MRI session (outside the scanner, in supine position, on the same day as the MRI). Following 5‐min of rest in the supine position, the mean of 10‐min of beat‐by‐beat data in supine position was used for analyses. The Finometer derives brachial BP from the reconstructed and level‐corrected finger blood‐flow waveform (Gizdulich, Imholz, van den Meiracker, Parati, & Wesseling, 1996). The Finometer is a reliable device for tracking BP in adults (Parati, Casadei, Groppelli, Di Rienzo, & Mancia, 1989), and the precision of BP measurement with this device meets the requirements of the American Association for the Advancement of Medical Instruments. Moreover, compared to other methods commonly used in clinical settings, the Finometer has the advantage of providing beat‐to‐beat BP time courses, which are frequently needed in our studies (Guelen et al., 2008; Syme et al., 2008; Syme et al., 2009). The heights of participants were also measured as a potential physiological influencer of PWV (Reusz et al., 2010).

### 2.2 MRI acquisitions

MRI data were acquired on a Philips Achieva 3.0 T scanner (Best, the Netherlands). The scans used eight‐channel phased‐array head coil reception and body‐coil transmission. A 3D T1‐weighted anatomical scan was acquired, with resolution 1 × 1 × 1 mm, repetition time (TR) = 4.7 ms, echo time (TE) = 2.3 ms, flip angle = 8°, field of view = 288 × 288 mm (sagittal), matrix size = 256 × 256, 164 slices, bandwidth = 479 Hz/pixel, and SENSE acceleration factor = 2.

Our rs‐fMRI data were acquired using a dual‐echo pCASL sequence (Dai et al., 2008). Detailed scanning protocols are as follows: TR = 4,000 ms, TE_CBF_/TE_BOLD_ = 10/30.7 ms, field of view = 220 × 220 mm, matrix size = 64 × 64, 20 slices (ascending interleaved order), voxel size = 3.44 × 3.44 × 6.0 mm^3^, the number of time frames = 100, bandwidth = 2,520 Hz/pixel, and SENSE acceleration factor = 2. The labeling duration was 1,500 ms, and the post‐labeling delay was 1,000 ms with a mean Gz of 1 mT/m. Slices were acquired in an ascending interleaved fashion.

Cardiac imaging was performed within the same scan session. First, an aortic localizer scan showing the “candy‐cane” view of the aorta was acquired for anatomical reference. Then, a retrospectively ECG‐gated gradient‐echo pulse sequence with velocity encoding (i.e., phase‐contrast MRI) was applied to measure through‐plane flow at two slice locations, one positioned through the aortic arch to quantify flow in the ascending and descending portions of the arch, and a second slice positioned in the abdominal aorta approximately 2 cm above the bifurcation. In line with previous work (Macgowan, Henkelman, & Wood, 2002), the relevant imaging parameters included: flip‐angle = 10°, TR = 4.9 ms, TE = 3.0 ms, views‐per‐segment (VPS) = 8, temporal resolution = 2 × TR × VPS, interpolated to ~25 ms during reconstruction, depending on heart rate, slice thickness = 8 mm, field of view = 350 mm, matrix size = 240 × 240, spatial resolution = 1.46 × 1.46 mm^2^ (interpolated to 1.25 × 1.25 mm^2^ during reconstruction), and Venc = 200 cm/s.

### 2.3 Preprocessing: fMRI data

The procedure used for preprocessing of the functional data is outlined in (Tak, Wang, Polimeni, Yan, & Chen, 2014). Specifically, the tag, control, and BOLD images in the dual‐echo pCASL data were separately preprocessed using SPM12 (Wellcome Trust Centre for Neuroimaging, London, UK) through the CONN toolbox (Whitfield‐Gabrieli & Nieto‐Castanon, 2012) (publicly available: https://www.nitrc.org/projects/conn). The first four time frames were discarded to ensure the MR steady state. Motion correction was performed using six‐parameter affine transformation, and slice‐timing correction was performed using sinc interpolation. All time frames were spatially normalized into the Montreal Neurological Institute (MNI) space and resampled to 2‐mm isotropic voxels. Spatial smoothing with a 6‐mm full‐width at half‐maximum (FWHM) Gaussian kernel was applied.

As the contributions of physiological noise to the interleaved tag and control images are different (Restom et al., 2006), the tag and control images were subjected to physiological noise correction separately. Anatomical segmentation was performed using FSL FAST's probabilistic tissue segmentation (Zhang, Brady, & Smith, 2001). The CSF and WM parcellations were used as noise regions of interest (ROIs), and their means signals regressed out the rs‐fMRI signal. After segmenting the T1‐weighted anatomical image into the different tissue classes, including gray matter (GM), WM, and CSF, we derived four significant principal components from the mean signal in the noise ROI using the aCompCor method (Behzadi & Liu, 2005). Physiological denoising within the tag and control images was performed separately. BOLD data were computed by performing surround averaging of the denoised control and tag images, whereas CBF data were computed by surround subtraction (Tak et al., 2014). Both time series were then band‐pass filtered to span 0.001–0.08 Hz, in keeping with the practice of targeting low‐frequency signal fluctuations in rs‐fMRI.

### 2.4 BOLD‐signal fluctuation maps

The voxel‐wise fluctuation amplitude of the BOLD time‐series generated from the preprocessing pipeline is computed as the variance of the signal using MATLAB (Mathworks, Natick), rejecting outliers. We used the anatomical tissue‐segmentations to mask the variance map and averaged the values to extract a mean GM and WM values of BOLD variance. The BOLD‐variance maps and the mean variance values in each ROI (i.e., WM, GM) were used throughout the analysis.

### 2.5 CBF fluctuation maps

As CBF is more directly related to neuronal metabolism than BOLD, we also calculated maps of fluctuation amplitude of the CBF series, defined as the variance of the signal, as was done for BOLD. Before this step, we were careful to minimize BOLD contamination of the CBF time series: the first echo of the pCASL time series was high‐pass filtered then demodulated to extract the CBF component, as is outlined in (Tak et al., 2014)). Due to the low reliability of WM CBF measurements using ASL, we did not extend the CBF analysis to the WM.

### 2.6 Quantitative CBF maps

To allow us to understand the link between potential PWV–BOLD relationships and perfusion status, we also generated quantitative CBF maps. We did so using the conventional pipeline (Chen, Rosas, & Salat, 2011) as well as using the ENABLE algorithm (Shirzadi et al., 2018). ENABLE is a multiparametric automated algorithm that offers the option of optimizing the signal‐to‐noise ratio (SNR) of the CBF time series by removing outlier time‐points. We generated both a default (no outlier removal) and an optimized quantitative CBF maps using ENABLE, with quantification obtained through the general kinetic model (Buxton et al., 1998).

### 2.7 Pulse‐wave velocity

**Figure 1:**
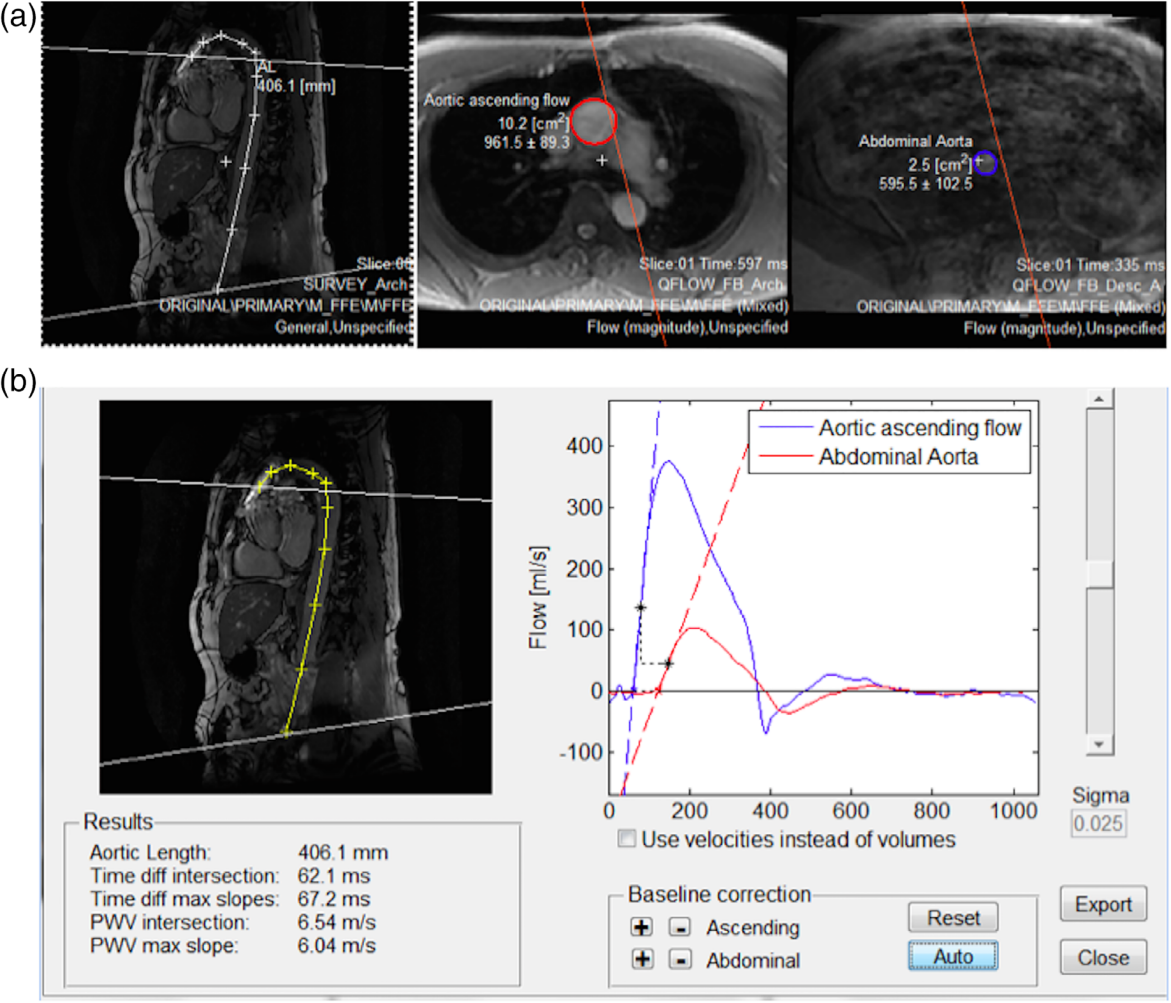
Sample PWV measurements. (a) Showing the flow ROI's and the intersecting planes in the oblique sagittal aorta image. (b) Example of the pulse wave velocity analysis window, showing the aorta length measurement and the flow curves

PWV was calculated using the time‐to‐foot approach (Dorniak et al., 2016), implemented through the freely available software Segment CMR (Medviso, publicly available at http://segment.heiberg.se) (Heiberg et al., 2010). Specifically, aortic PWV was calculated as *x*/*t* (expressed in m/s), where *x* is the aortic path length between the arch and abdominal aorta, and *t* is the time delay between the arrival of the foot of the pulse wave at these locations. The aortic length is computed as the distance between the arch and the abdominal aorta, each labeled manually in the Segment CMR package, as shown in Figure 1. PWV estimates were obtained by two independent raters and averaged to generate the final PWV values. In our group, the mean aortic length was 35.6 ± 3.2 cm. The inter‐rater correlation of PWV estimates is 0.81, with an *r* ^2^ of 0.70.

### 2.8 Cortical‐thickness measurements

We performed cortical‐surface reconstruction using FreeSurfer (publicly available at http://surfer.nmr.mgh.harvard.edu). The procedure includes removal of non‐brain tissue using a hybrid watershed/surface deformation procedure (Segonne et al., 2004), automated transformation into the MNI152 standard space, intensity normalization (Sled, Zijdenbos, & Evans, 1998), tessellation of the GM WM boundary, automated topology correction (Segonne, Pacheco, & Fischl, 2007), and surface deformation following intensity gradients to optimally place the gray/white and gray/CSF borders at the location where the greatest shift in intensity defines the transition to the other tissue class (Fischl & Dale, 2000). The subsequent segmentation of the cortex and subcortical GM volumetric structures were performed for each subject based on probabilistic models of tissue magnetic resonance parameters and of anatomical locations (Fischl et al., 2004). The resultant cortical models permitted the calculation of cortical thickness maps as well as surface inflation (Fischl et al., 1999) and registration to a spherical atlas, whereby individual cortical folding patterns were used to match cortical geometry across subjects (Fischl, Sereno, & Dale, 1999).

### 2.9 Role of vasculature: vascular‐probability maps

Because PWV is an established metric for assessing macrovascular stiffness, we are interested in potential links between probability of macrovascular occupancy and the observed PWV, BP effects in rs‐fMRI. Thus, a digital vascular‐frequency atlas (Viviani, 2016) was used for reference (publicly available: https://www.uniklinik-ulm.de/psychiatrie-und-psychotherapie-iii/forschung-studien/clinical-neuroimaging.html). This atlas was constructed from time‐of‐flight (TOF) data obtained with MR angiography in 38 healthy adults. Specifically, the individual TOF data were thresholded to 97 percentile of signal intensity to allow for localization of major blood vessels (arteries and veins) and spatially registered to the MNI152 space at a 1 mm isotropic resolution. The quantities in this map represent the percentage of individuals manifesting a mid‐to‐large size blood vessel at a given voxel (i.e., vascular probability map). These quantities are then summarized into equal‐sized bins to help observe general trends.

Furthermore, we manually segmented out arterial and venous subsets of the vessel atlas. The arterial map consists of the Circle of Willis, the anterior and middle cerebral arteries, and the cingulate artery. The venous map consists of the superior and inferior sagittal sinuses, the straight sinus, the transverse sinus, and the Great Vein of Galen. We were not able to extend this separation to the smaller arteries and veins, which are more entangled and less discernible. Also, the arterial versus venous vascular probabilities are not binned.

### 2.10 Functional connectivity: intrinsic connectivity contrast maps

To determine the influence of PWV (and related variables) on resting‐state functional connectivity, we computed intrinsic connectivity (IC) contrast maps for each participant (Constable, Hara, Tokoglu, & Papademetris, 2009; Martuzzi et al., 2011). First, for each voxel, a correlation analysis is performed between its time series and that of every other voxel in the brain volume. Then, the correlation coefficients are converted to *z* scores via the Fisher transform and summed, for a given “seed” voxel, across all correlated voxels. The IC value at each “seed voxel” is defined as this sum, and this calculation is repeated at each voxel. The IC map provides a network‐independent view of brain connectivity. That is, each IC value represents how strongly the given voxel is “connected” with the rest of the brain, and it is based on similar principles as conventional seed‐based connectivity analysis.

### 2.11 Statistical analyses

#### 2.11.1 Mixed‐effects modeling

To identify potential covariations between PWV and other variables, we constructed a linear mixed‐effects model using MATLAB. The fixed‐effects considered include height, age, SBP, DBP, and pulse pressure (PP), with sex as a grouping variable. All of these have been linked to PWV values (Reusz et al., 2010; Smulyan et al., 1998). The model can be summarized by Equations (1)–(3).

(1) $$\mathrm{PWV}\sim 1+\mathrm{age}+\text{height}+\mathrm{SBP}+\left(1|\mathrm{sex}\right)$$

(2) $$\mathrm{PWV}\sim 1+\mathrm{age}+\text{height}+\mathrm{DBP}+\left(1|\mathrm{sex}\right)$$

(3) $$\mathrm{PWV}\sim 1+\mathrm{age}+\text{height}+\mathrm{PP}+\left(1|\mathrm{sex}\right)$$

The model includes an intercept that accounts for random inter‐individual differences not accounted for by the fixed effects.

#### 2.11.2 General‐linear model analyses

As an initial examination of the data, we assessed the relationship between PWV and mean BOLD variance in all GM across participants. The same was done for mean BOLD variance in WM. We did not observe significant associations in either case, leading to the voxel‐wise tests. We further divided the group into an offspring (age = 19–39) and a parent (age = 44–67) group, which was convenient given the bi‐generational design. We repeated the PWV‐related GLM analyses in each age group.

Voxel‐wise correlations were performed between PWV and the BOLD variance maps across participants. Statistical significance was performed using FSL randomize (https://fsl.fmrib.ox.ac.uk/fsl/fslwiki) with cluster thresholding using threshold‐free cluster enhancement (TFCE), corrected for multiple comparisons using a 10,000‐iteration permutation testing (Winkler, Ridgway, Webster, Smith, & Nichols, 2014). The same method was used to assess the effect of potential explanatory variables, including age, SBP, and DBP. Furthermore, in a multivariate GLM, we tested for the effect of PWV on the BOLD metrics while including age as a covariate. This process was repeated for the quantitative CBF and CBF signal‐variance maps. Further statistical testing was performed with age included as a covariate.

Furthermore, we correlated the vascular probability against the statistical significance of the BOLD‐PWV association to assess the potential links between macrovascular content and BOLD‐susceptibility to PWV influence. As the vascular probability maps are quantized (by definition), we binned their values into equidistant bins and used the bin‐averages in the regression analysis. We further distinguished the maps into large‐artery and large‐vein probability maps and repeated the linear‐regression analysis.

Furthermore, we performed surface‐based GLM analysis to assess the extent to which the BOLD‐PWV effects are driven by concurrent PWV‐tissue‐volume effects. This is done using FreeSurfer mri_glmfit, whereby cortical thickness was included as a covariate. BOLD and CBF‐variance maps were, respectively, registered to their corresponding T1‐anatomical volumes using bbregister. Subsequently, brain‐surface models (see Cortical‐thickness analysis) from all subjects were registered to the fsaverage space using spherical registration. The effect across sexes was averaged.

#### 2.11.3 Mediation analysis

We performed mediation analyses to more explicitly assess the contribution of age and CBF fluctuations to the observed BOLD‐PWV associations. To assess the significance of the mediation effects, we used the bootstrapping based method described by Preacher et al. (Preacher, Rucker, & Hayes, 2007) and implemented in the BRAVO Toolbox (publicly available at https://sites.google.com/site/bravotoolbox/). As inputs we used BOLD variance, CBF variance and mean CBF values derived as averages over relevant ROIs, and we bootstrapped for 1,000 iterations.

## 3 RESULTS

### 3.1 PWV mixed‐effects modeling

**Figure 2:**
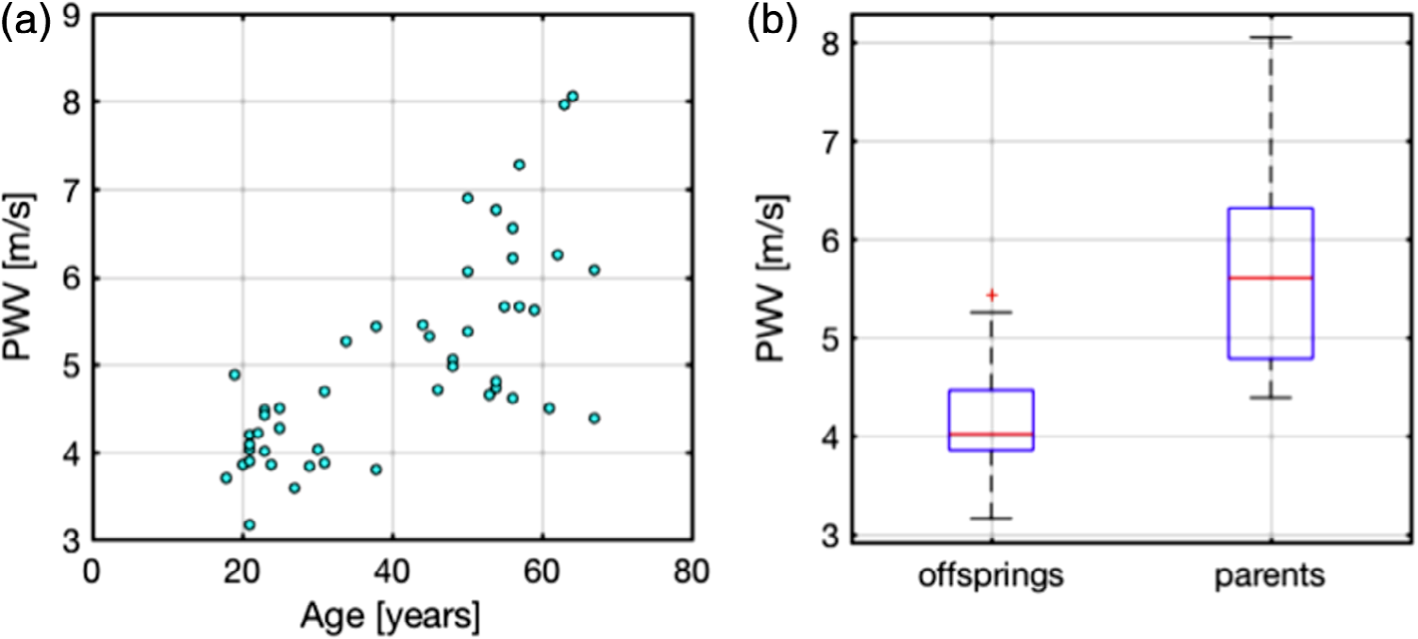
PWV–age associations. (a) PWV is shown over the age range of our cohorts, with no clear group‐wise age gap between our parent and offspring groups. (b) On average, the PWV of the parent group is higher than that of the offspring group (*p* < 0.01)

The mean PWV of the parent and offspring groups were 5.74 ± 1.05 and 4.17 ± 0.53, respectively. The group comparison and age dependence are illustrated in Figure 2. The mixed‐effects model showed that PWV is associated with age (*p* = 4.01 × 10^−9^) and systolic BP (SBP, *p* = 0.02). No association was found between PWV and diastolic BP (DBP; *p* = 0.17), PP (*p* = 0.07), height (*p* = 0.23), or sex (*p* = 0.33). For this reason, we only examined the associations between resting BOLD variance and age as well as systolic BP, in addition to PWV.

### 3.2 BOLD fluctuation amplitude versus PWV

**Figure 3:**
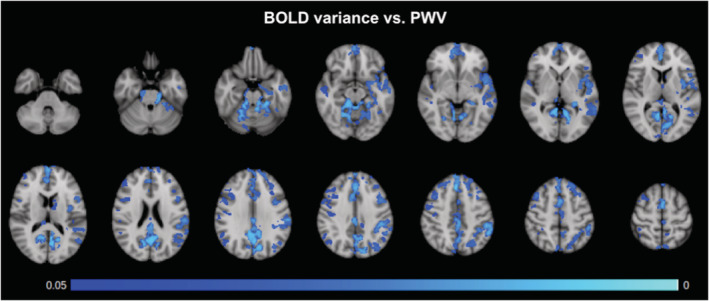
BOLD‐signal fluctuations (variance) versus PWV. *p*‐value map of regions with negative relationship between PWV and BOLD signal fluctuation, corrected for multiple comparisons and thresholded at *p* < 0.05. The primary clusters found are in the precuneus, anterior and posterior cingulate gyrus, paracingulate gyrus, and frontal pole as well as the temporal pole and parahippocampal regions

Although the mean‐global amplitude of the resting BOLD fluctuations (i.e., BOLD variance) was not significantly associated with PWV, we observed region‐specific associations between BOLD variance and PWV (Figure 3). All statistical maps are thresholded at *p*_Corrected_ < 0.05. The prominent areas exhibiting this relationship were the precuneus, the anterior and posterior cingulate gyrus, the paracingulate gyrus, and the frontal pole as well as the temporal pole, parahippocampal region. There were no significant associations in the WM. Also, after correction, there were no significant associations were found between PWV and quantitative CBF, nor was there a significant relationship with CBF variance.

When looking at each age group individually, the offspring and parent group PWV values were 4.14 ± 0.53 and 5.74 ± 1.05 m/s, respectively. We noted that the mean‐GM BOLD fluctuation variance in the young (offspring) group was not associated with PWV (*p* = 0.52), whereas the association was negative in the older (parent) group (slope = −0.32, *p* = 0.02). When regressing out cortical‐thickness variations with PWV, we observed minimal changes in the BOLD‐PWV associations (see Figure A2, Supplementary Materials).

**Figure 4:**
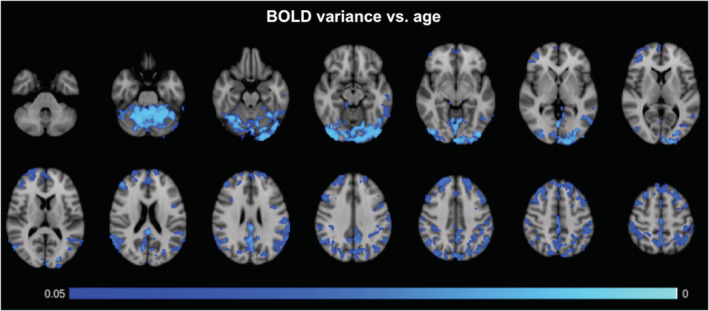
BOLD‐signal fluctuations (variance) versus age. Negative associations between age and BOLD variance, FWE‐corrected for multiple comparisons and thresholded at *p* ≤ 0.05. Primary clusters are found in the cerebellum, the posterior cingulate gyrus, and precuneus. The age effects are also widespread in the superior parietal and frontal regions

As part of a secondary analysis, we observed region‐specific associations between resting BOLD fluctuation amplitude (i.e., BOLD variance) and age (Figure 4). Once again, all statistical maps are thresholded at *p*_Corrected_ < 0.05. The age‐effects are found in the cerebellum, posterior cingulate gyrus, precuneus, as well as large regions of the superior parietal and frontal cortices. Once again, neither significant association was found between age and quantitative CBF, nor was there a significant relationship with CBF variance. Moreover, no significant association was found between SBP and the BOLD variance, CBF variance, or quantitative CBF. However, when averaging over the entire region that demonstrated significant association between BOLD variance and PWV, we found a significant relationship between mean CBF variance and PWV as well (*p* = 7.1 × 10^−5^), suggesting that our inability to observe significant voxel‐wise association may be due to noise.

**Figure 5:**
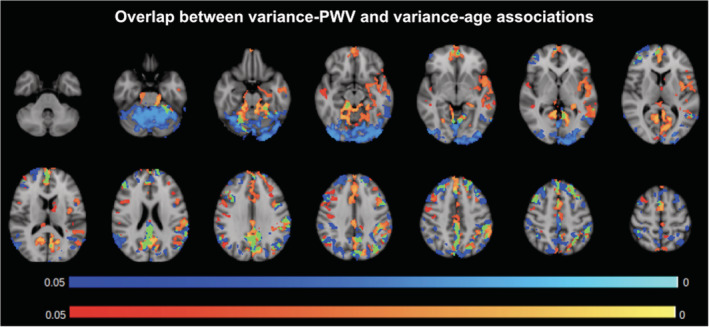
Comparison of BOLD–PWV and BOLD‐age associations. *p*‐value maps of BOLD and PWV negative correlations (orange) and BOLD and age negative correlations (blue), FWE‐corrected for multiple comparisons and thresholded at 0.05. Regions of overlap are shown in green, and the color bars illustrate the corrected *p*‐values

A natural next question was how much PWV effects account for age effects. In Figure 5, we illustrate the spatial association and dissociation between the PWV effects and age effects on BOLD signal variance. Although PWV is shown to be associated primarily with age in the mixed‐effects analysis, the two effects overlap mainly in the medial‐temporal, medial frontal, and precuneus/posterior cingulate regions. The temporal pole, parahippocampal region, and anterior cingulate region, which are affected by PWV (orange), are not affected by age (blue). Conversely, the occipital lobe and superior parietal lobule, which are associated with age, are not associated with PWV. No significant effects could be attributed to SBP or DBP.

**Figure 6:**
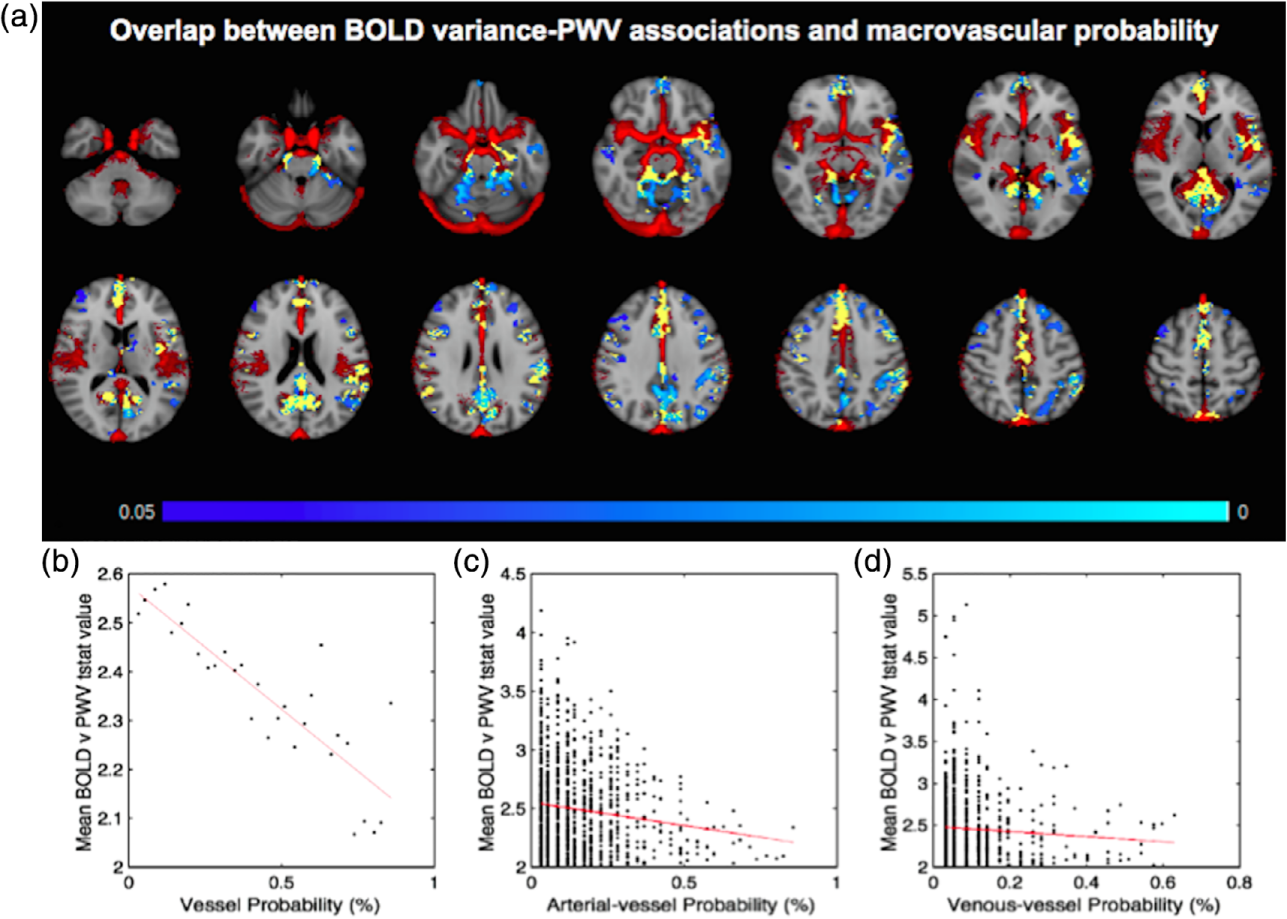
Macrovascular contribution to BOLD–PWV associations. (a) *p*‐value map showing BOLD and PWV negative correlations (blue) FWE‐corrected for multiple comparisons and thresholded at 0.05, and a vessel frequency atlas showing the frequency of detection for large vessels (red). Regions of overlap are shown in yellow. (b) The vessel probability values are binned into 30 bins, and each symbol in the plot represents the average across all voxels with the given bins. The best linear fit of the relationship is represented by the red line. The same relationship is shown for arterial vessels (c) and venous vessels (d), without binning in these latter cases. The main finding is that the highest BOLD–PWV associations (*t* scores) tend to be found in regions of low large‐vessel probability, whether it be arteries or veins

Furthermore, in terms of the co‐localization of the observed BOLD‐variance effects and the macrovasculature, 45% of the voxels demonstrating a BOLD association with PWV are found in regions with major blood vessels. This was computed as the percentage of overlap between vascularized voxels (indicated by the vascular atlas) and those shown a significant BOLD‐PWV association. As can be seen in Figure 6a, the vascular‐frequency map (red) clearly delineates major arteries and veins, including the Circle of Willis, the superior sections of the middle‐cerebral artery, the superior sagittal sinus, and transverse sinus.

A more in‐depth examination of these effects entailed obtaining the mean t‐stat values from significant voxels following the group analysis. That is, we extracted voxel‐wise t‐values from regions exhibiting significant PWV associations with BOLD variance (MNI space), as well as the corresponding vessel probability values. The voxel‐wise t‐stat values are plotted against the voxel‐wise vascular probability values (Figure 6b–d). The relationship is also assessed in manually demarcated arterial and venous sections of this vascular atlas. The results suggest no difference in the BOLD‐PWV association between arterial and venous regions.

### 3.3 Functional connectivity versus PWV

**Figure 7:**
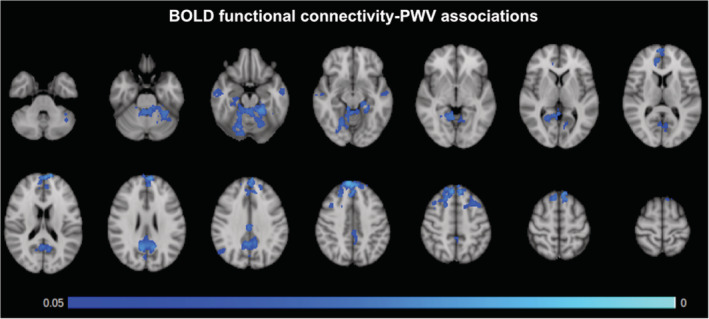
Associations between BOLD functional connectivity and PWV. *p*‐value map of the negative correlation between intrinsic connectivity contrast values and PWV, FWE‐corrected for multiple comparisons and thresholded at 0.05. Primary regions include the precuneus cortex and the frontal lobe

As seen in Figure 7, IC values are negatively associated with PWV and in the same general areas that demonstrated a negative association between BOLD variance and PWV (Figure 3). Namely, the cortical regions in which higher PWV is associated with lower functional connectivity include the precuneus, posterior cingulate, and medial prefrontal cortex. However, there was no significant association between IC and age. No significant effect of SBP and DBP were identified.

**Figure 8:**
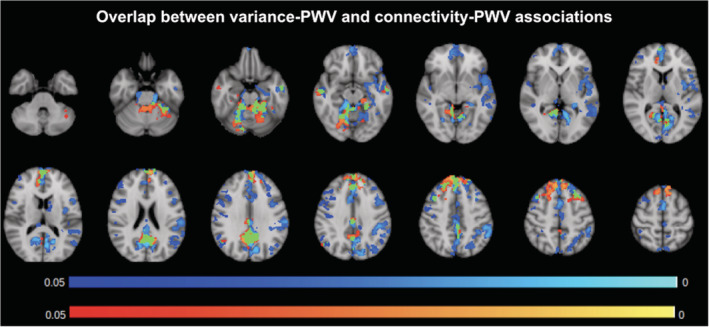
Comparison of BOLD–PWV and connectivity–PWV associations. *p*‐value maps of BOLD‐variance‐PWV associations (blue) and IC‐PWV associations (orange), FWE‐corrected for multiple comparisons and thresholded at 0.05. Regions of overlap are shown in green. The overlap in effects are observed in the medial‐temporal, medial frontal, and precuneus/posterior cingulate regions

The similarities and differences of the group‐wise PWV effects on BOLD‐variance and IC are summarized in Figure 8. The effects overlap in the medial‐temporal, medial frontal, precuneus, as well as posterior cingulate regions. However, the majority of the effects do not overlap. That is, although the BOLD variance‐PWV associations are unique (not overlapping with IC effects) to the medial‐orbitofrontal, paracentral, superior temporal, lateral‐superior frontal, and superior parietal regions, the IC‐PWV associations are unique to the superior and middle frontal gyri.

The results from the mediation analysis are summarized in Table 2. In brief, when averaging across all voxels exhibiting a significant association between BOLD variance and PWV, CBF variance (fluctuation amplitude) is found to be a significant mediator of BOLD‐variance‐PWV associations, but not basal mean CBF. Also, when averaging over all voxels exhibiting significant associations between BOLD variance and age, PWV is found to be significant mediator (*p* = 4.9 × 10^−4^). Finally, BOLD variance is found to be a significant mediator of the associations between FC and PWV.

**Table 2:** Mediation analysis results, demonstrating the mediation effects of (1) PWV on the associations between BOLD variance and age; (2) CBF fluctuations on the association between BOLD variance and PWV. In each case, the values are taken as ROI means. BOLDvar = BOLD variance, CBFvar = CBF variance, meanCBF = quantitative CBF

Effect	Region of interest (ROI)	*p*‐values
PWV mediation of BOLDvar–age association	Significant BOLDvar–age association	4.9 × 10^−4^
Age mediation of BOLDvar–PWV association	Significant BOLDvar–PWV association	9.3 × 10^−5^
CBFvar mediation of BOLDvar–PWV association	Significant BOLDvar–PWV association	6.6 × 10^−5^
meanCBF mediation of BOLDvar–PWV association	Significant BOLDvar–PWV association	0.49
BOLDvar mediation of FC–PWV association	Overlap of significant BOLDvar–PWV association and significant FC–PWV association	2.8 × 10^−4^

## 4 DISCUSSION

The role of vascular stiffness as a risk factor for age‐related cognitive deficits is being actively investigated (Scuteri, Brancati, Gianni, Assisi, & Volpe, 2005; Tarumi et al., 2013; Tsao et al., 2013). As mentioned earlier, arterial stiffness could simultaneously affect CBF, CVR, and blood pressure, all of which are known to affect the fMRI signal, during tasks and in resting state (Chu et al., 2018; Golestani et al., 2016; Liu, 2013; Wang et al., 2006). However, there has been very limited research into the relationship between vascular stiffness and imaging‐based “functional” measures, particularly those obtained using rs‐fMRI. In this study, in our sample of healthy young to middle‐aged adults, we find that: (1) there is a significant negative association between PWV and rs‐fMRI low‐frequency BOLD fMRI signal amplitude; (2) these associations are spatially distinct from the effect of age on resting‐BOLD signal ampoitude (which are also negative); (3) PWV is negatively associated with resting‐state functional connectivity. All of our hypotheses are confirmed by the experimental data. Furthermore, all of these effects are most robustly observed in the medial‐temporal, medial frontal, and precuneus/posterior cingulate regions.

### 4.1 BOLD fluctuation amplitude versus PWV

Although the brain is supplied by the carotid arteries and not directly by aortic flow, the established correlation between aortic and carotid PWV (measured between two points of the internal carotid artery) (Nagai et al., 1999) enables us to use aortic stiffness as a surrogate for carotid stiffness. Vascular stiffness has been associated with impaired cognitive‐task response as measured using near‐infrared spectroscopy (Vermeij, den Abeelen ASS, RPC, AHEA, & JAHR, 2014) and reduced fMRI response to a working‐memory task as measured using BOLD (Steward et al., 2014).

A main determinant of the BOLD signal amplitude is cerebrovascular reactivity (CVR). CVR is reported to be reduced by arterial stiffening in clinical cases (Edwards, 2006), and a related measure, cerebrovascular conductance is also found to be inversely correlated with PWV (Jaruchart, Suwanwela, Tanaka, & Suksom, 2016). By definition, increased vascular stiffness translates into reductions in vascular elasticity (Kotsis & Stabouli, 2011; Zavoreo & Demarin, 2010), hence reduced CVR and reduced BOLD signal amplitude. Such associations between CVR and the resting‐state BOLD signal have been reported previously by ourselves and others (Chu et al., 2018; Golestani et al., 2016; Kannurpatti, Motes, Biswal, & Rypma, 2014). Our finding of decreasing resting BOLD signal variance with increasing PWV is consistent with task‐based findings and supports our hypothesis the common mediator may be diminishing vascular reactivity. These findings extend the influence of arterial stiffening to the resting brain. In theory, by the same token, we expect the same PWV associations with dynamic CBF fluctuations, but our data sample did not allow us to uncover such effects.

Given the relative large voxel sizes used in fMRI studies, it is possible that our results are affected by partial‐volume effects that evolve with tissue atrophy. Indeed, as mentioned earlier, cortical atrophy has been associated with high PWV (Pasha et al., 2015). However, our results (Figure A2) demonstrate otherwise. In fact, although we confirm widespread cortical‐thickness associations with PWV—high PWV associated with low thickness, there is minimal overlap between thickness associations and BOLD variance associations. Thus, controlling for thickness did not alter our findings.

Our linear mixed‐effects model indicates that age is the strongest predictor of PWV, followed by BP and height. This is in agreement with prior findings by others (Reference Values for Arterial Stiffness' Collaboration, 2010). Thus, our second hypothesis is that the PWV effects on the BOLD signal should be somewhat similar to those of age. However, our experimental findings demonstrate that age effects are distinct from those of PWV. As seen in Figure 5, BOLD signal amplitude in the temporal pole, parahippocampal region, and anterior cingulate region are uniquely associated with PWV, whereas BOLD signal in the occipital lobe and superior parietal lobule are uniquely associated with age.

Although age is a main risk factor for arterial stiffening, it is also a complex metric that is difficult to interpret physiologically. Different individuals of the same age may have very different vascular‐health status. It follows that age is not the one single strongest predictor of PWV (Magalhães et al., 2013), and PWV is a predictor of stroke occurrence independently of age (Laurent et al., 2003). Moreover, age may not be linearly related to PWV as is widely assumed (Tarumi et al., 2014). Nonetheless, the mediation analysis showed that age‐BOLD associations are significantly mediated by PWV (Table 2). Further analysis of a larger sample size is required to disentangle the causality between age and PWV effects on the BOLD signal.

In this study, no association among blood pressure (SBP or DBP), pulse pressure (PP = SBP − DBP), and rs‐fMRI metrics were found. This is contrary to expectation, based on past findings that blood pressure is a well‐established covariate of PWV (Gesche, Grosskurth, Küchler, & Patzak, 2012; Waldstein et al., 2008), and specifically, systolic BP has been suggested as a surrogate of PWV in healthy conditions (Gesche et al., 2012). There has also been suggestion that DBP is more closely linked with PWV in hypertension (Ni, Wang, Hu, & Zhang, 2003). However, these findings are dependent on the choice of cohort and the means of measuring BP. In healthy adults, BP may only affect brain function through PWV (Hajjar, Goldstein, Martin, & Quyyumi, 2016), as PWV was found to be a strong mediating factor in how BP affects executive function. This is echoed by our data, which shows only a moderate relationship between PWV and BP (SBP and DBP), with PWV explaining more of the rs‐fMRI variations than either BP measures. In addition, our ability to model the BP–PWV relationships as a function of factors such as age and sex is limited due to our moderate sample size.

An interesting finding is that the voxel‐wise macrovascular contribution to the BOLD signal is inversely correlated with the strength of the BOLD‐PWV association (Figure 7). That is, the highest BOLD‐PWV associations (*t* scores) tend to be found in regions of low large‐vessel probability, whether it be arteries or veins. This finding is reminiscent of our previous finding that the BOLD‐CBF coupling strength is inversely associated with macrovascular contribution (Tak et al., 2014), and that functional‐network strengths are strongest away from large vessels (Tak, Polimeni, Wang, Yan, & Chen, 2015). In the brain, the stiffening‐related increase in perfusion pressure is typically compensated by increased local blood‐flow resistance at the microvascular level, which, in the long term, would lead to macrovascular remodeling (including vascular and lumen enlargement as well as reduced vascular elasticity) (Mitchell, 2008). Thus, the utility of PWV monitoring does not stop at the level of the macrovasculature, at least not in the brain. However, the exact macro‐ versus microvascular mechanisms underlying this finding requires further investigation. Moreover, there was no difference in arterial and venous contributions to macrovascular‐density effect, as shown in Figure 6b–d. This may appear counterintuitive, given that we expect the BOLD signal at 1.5 T to be largely driven by venous blood. However, as our recent work shows, arterial‐blood volume also contributes to the resting‐state BOLD signal (Khajehim & Chen, 2018a; Khajehim & Chen, 2018b).

Another main determinant of BOLD signal amplitude is CBF, and this occurs in two ways. First, based on the BOLD biophysical model (Davis, Kwong, Weisskoff, & Rosen, 1998; Hoge et al., 1999), dynamic CBF fluctuations directly drive BOLD signal fluctuations. Second, the amplitude of the BOLD fluctuation is in theory modulated by the underlying baseline CBF. Interestingly, at a voxel‐wise level, the observations made on the BOLD signal are not echoed by the dynamic CBF signal. Moreover, although work by Tarumi et al. showed that higher carotid‐femoral PWV was associated with lower CBF in frontal and parietal WM (Tarumi et al., 2011), this was not observed in our data, potentially due to the more stringent exclusion criteria employed in this study. In the Tarumi study, those with controlled hypertension and diabetes mellitus were not excluded, whereas they were not part of the current cohort. As our previous work showed (Tak et al., 2015), BOLD and CBF are not tightly coupled in all voxels in the resting state. Moreover, CBF data is inherently noisier than its BOLD counterpart, and in the attempt to uncover significant PWV associations, we may be limited by the sampling interval, the number of frames, and the number of subjects. Nonetheless, when averaging across all voxels exhibiting a significant BOLD‐PWV association, mean CBF variance is found to be significant mediator of this association (Table 2), which is an encouraging finding.

In relation to the BOLD or CBF signal fluctuation in the WM or deep GM, we did not observe any effect of PWV. This may be due to the fact that the WM region was used to extract noise regressors that were subsequently removed from the BOLD signal. Furthermore, ASL reliability in the WM is lower than in GM. Therefore, we are unable to interpret the findings in the WM. Notably, the contribution of physiological noise in periventricular GM (i.e., basal ganglia) is well established (Behzadi & Liu, 2005; Chang, Cunningham, & Glover, 2009). Therefore, our intuition is that the deep‐gray BOLD signal quality is poorer than in the cortex and may be more strongly driven by non‐brain mechanisms despite physiological denoising.

### 4.2 Functional connectivity versus PWV

Our findings of reduced functional connectivity with higher PWV are consistent with the work of Guevara et al. (Guevara et al., 2013), although in the latter case arterial stiffening was chemically induced in mice. Although PWV is strongly associated with age, as revealed by our linear mixed‐effects model (*p* = 4.0 × 10^−9^), age is not significantly associated with connectivity as represented by IC. Our findings suggest that the effect of PWV on rs‐fMRI are unique from those of aging, and that with regards to connectivity, PWV has stronger influence than does age. In fact, although the age effect is likely an aggregate of multiple factors, only a subset of which are investigated here (i.e., PWV, blood pressure, stroke volume), our findings suggest that vascular effects may have a dominating influence in age‐connectivity associations. This viewpoint is further bolstered by the significant mediating effect of PWV in BOLD‐age associations (Table 2).

The effect of PWV on BOLD‐signal variance and on IC overlap in all main nodes of the default‐mode network (DMN), namely the medial‐temporal, medial frontal, precuneus, as well as posterior cingulate regions (Figure 8). Recall that the IC is derived as the sum of all voxel‐wise correlations. It is expected, therefore, that effects in the BOLD‐signal variance do not translate into effects on the IC. The differences in the localization in the BOLD variance‐IC effects cannot be attributed solely to systematic contrast‐to‐noise differences between the two measures, as each measures exhibit unique sensitivity to PWV. Rather, parallels between BOLD‐signal amplitude and corresponding connectivity trends can only be drawn in the DMN, but not anywhere else. Existing literature points to the likelihood that brain regions with high metabolic demand are most prone to the effect of pulse‐wave related damage (Lamballais et al., 2018). Evidence from this study also suggest that a combination of BOLD‐signal variability and BOLD‐based connectivity providing a sensitive marker of PWV‐related brain changes.

Although it is intuitive to attribute the effects in IC solely to those in BOLD‐fluctuation amplitude, this reasoning is not supported by the data (Figure 8). In fact, the relationship between correlation‐based functional connectivity and BOLD‐signal amplitude is rather complex (Chu et al., 2018). Our previous work shows that, reductions in CVR (in this case a common result of arterial stiffening) will be reflected in reduced BOLD‐signal variance but not necessarily into reduced functional correlations, as the latter is also dependent on basal CBF. Not having found significant links between PWV and basal‐mean CBF in our study, we may expect that the regions showing reduced IC with increasing PWV to be a subset of those showing reduced BOLD‐signal variance. However, this is not the case. In fact, IC is uniquely reduced in the superior and middle‐frontal gyri, which do not show BOLD‐signal variance effects. Thus, the next logical question is the role of physiological noise in the IC scores. Physiological noise may affect the observed functional connectivity without modulating the BOLD signal amplitude, and the identification of such noise is non‐trivial and beyond the scope of this work.

It bears mentioning that the BOLD signal is a neurovascular signal. Sources of signal change include: (1) neurovascular coupling; (2) vasomotion; (3) cardiac pulsation and cardiac variability (resulting in regional pulsatile motion); (4) respiration and respiratory variability (resulting in head motion and local‐field variations); and (5) head motion (unrelated to physiological effects). These effects are not the theoretical BOLD effect, but are nonetheless captured in the gradient‐echo EPI BOLD signal. Although we are certain that PWV can be associated with non‐oxygenation related changes in the BOLD signal, the current data do not allow us to make any definitive claims. It suffices to state that if the non‐neuronal effects are captured in the BOLD signal, then they may well be captured in the functional‐connectivity measures. The neuronal and vascular constituents of functional connectivity measurements themselves are complex to decipher and also beyond the scope of this work.

### 4.3 Limitations

A limitation of our data‐acquisition approach is the use of dual‐echo pCASL, which restricted our minimum‐achievable TR to 4 s. This in turn limited the number of time points that could be involved in the BOLD‐variance and IC calculations. However, the duration of the scan (~6 min) is typical of rs‐fMRI studies; thus we believe the findings are generalizable. This remains to be confirmed experimentally using highly sampled fMRI data, in which the effects of aliased cardiac and respiratory cycles can be better controlled. Of course, the use of pCASL is also strength of this study, as it gave us the possibility of obtaining static and dynamic CBF information. These metrics are complementary to the BOLD metrics and allowed for clarification of the origins of the BOLD‐related observations.

Also related to the acquisition is the fact that no physiological recording was obtained during the scans. Although the lack of physiological recordings is not uncommon in the field of rs‐fMRI, such recordings could enable future investigations of the relationship between cardiac pulsation and PWV.

Another potential limitation is the size of our cohort. Contrary to our hypothesis, we did not observe significant associations between CBF and PWV at a regional level. We intend to verify this pilot finding in a larger cohort.

## 5 CONCLUSION

In conclusion, in a group of healthy young to middle‐aged adults, we demonstrate that rs‐fMRI signal amplitude and the resulting functional connectivity are significantly and inversely related aortic pulse‐wave velocity. These effects are also mediated by age. These findings introduce a new dimension into the interpretation of rs‐fMRI as a presumed means to measure neural activity and brain networks. In particular, the findings of this study should be taken into account in rs‐fMRI studies of aging.

## CONFLICT OF INTERESTS

The authors declare no conflict of interest.

## Supporting information


**Appendix** **S1**: Supplementary Materials

## Data Availability

Data can be made available upon request to T. Paus.
