# Supplementary material for: The association between resting‐state functional magnetic resonance imaging and aortic pulse‐wave velocity in healthy adults
Source: Hum Brain Mapp. 2020 Feb 7;41(8):2121–35. doi: 10.1002/hbm.24934 (PMC7268071; doi:10.1002/hbm.24934)
Supplement: Supplementary file 1 — Appendix S1: Supplementary Materials [file HBM-41-2121-s001.docx]

Supplementary Materials


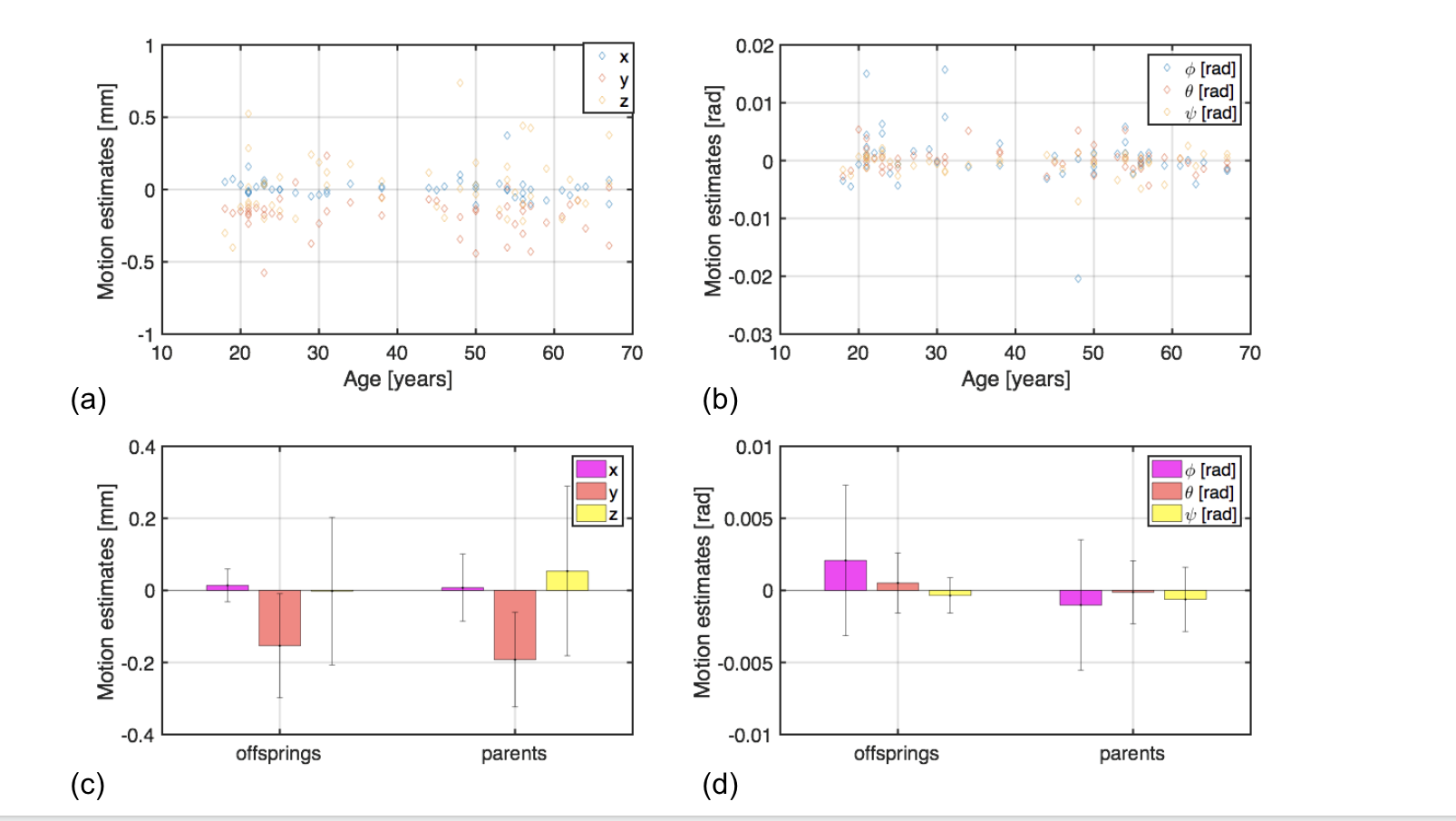


**Figure A1. The age dependence of motion estimates.** (a) The translational motion estimates across ages. Each symbol indicates one subject and one parameter. (b) The rotational motion estimates across ages. (c-d) Despite the strong association between PWV and age, there is no significant age dependence found in motion estimates, either translation or rotation.

**
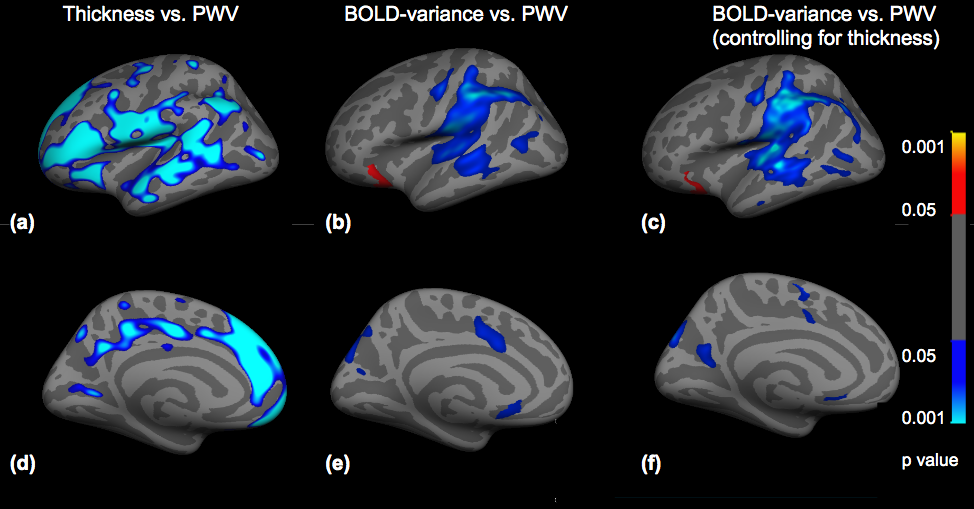
Figure A2. The relationship between cortical-thickness and PWV, and its contribution to the BOLD-PWV association.** The lateral and medial surfaces are shown for each measure. While cortical thickness is strongly and negatively associated with PWV (a & d), these associations for the most part do not overlap with the BOLD-variance-PWV associations (b & d). In fact, controlling for cotical-thickness associations did not alter the BOLD-variance-PWV associations (c & f).
